# Wearing the Beard

**Published:** 1854-01

**Authors:** 


### *Wearing the Beard.*

Although it is not customary to wear the beard in the United States, it would undoubtedly contribute to the health of men to do so, at least if unshorn from the edge of the jaws and chin downward. Clergymen would in all cases be benefited by it. Hair is nature's protector against cold. Our beneficent Creator does nothing in vain.

Rowland says on this subject,

"It may be safely argued as a general physiological principle, that whatever evinces a free and natural development of any part of the body, is, by necessity, beautiful. Deprive the lion of his mane, the cock of its comb, the peacock of the emerald plumage of its tail, the ram and deer of their horns, and they not only become displeasing to the eye, but lose much of their power and vigor. And it is easy to apply this reasoning to the hairy ornaments of a man's face. The caprice of fashion alone forces the Englishman to shave off those appendages which give to the male countenance that true masculine character, indicative of energy, bold daring, and decision. The presence or absence of the beard, as an addition to the face, is the most marked and distinctive peculiarity between the countenances of the two sexes. Who can hesitate to admire the noble countenance of the Osmanli Turk of Constantinople, with his un-Mongolian-length of beard? Ask any of the fair sex whether they will not approve and admire the noble countenance of Mehemet Ali, Major Herbert Edwards, the hero of the Punjaub, Sir Charles Napier, and others, as set off by their beard? We may ask, with *Beatrice*, 'What manner of man is he? Is his head worth a hat, or his chin worth a beard?' I have noticed the whiskers and beards of many of our most eminent physicians and merchants encroaching upon their former narrow boundaries, while it is well known that not a few of our divines have been long convinced of the folly of disobeying one of nature's fixed laws; but hitherto their unwillingness to shock the prejudice of their congregations, have prevented them from giving effect to their convictions. The beard is not merely for ornament, it is for use. Nature never does anything in vain; she is economical, and wastes nothing. She would never erect a bulwark were her domain unworthy of protection, or were there no enemy to invade it."

### *Eyelashes.*

In Circassia and neighboring countries the eyelashes of children are clipped, with scissors, at their extreme points, while asleep, every six weeks, giving them in time a beautiful gloss and curve, besides adding to their length and thickness.

